# Authors’ Reply: Balancing Challenges and Opportunities When Evaluating Remote Rehabilitation for Sarcopenia in Older Adults

**DOI:** 10.2196/73174

**Published:** 2025-04-01

**Authors:** Lu Zhang, Ying Ge, Wowa Zhao, Xuan Shu, Lin Kang, Qiumei Wang, Ying Liu

**Affiliations:** 1 Department of Rehabilitation Medicine Peking Union Medical College Hospital Chinese Academy of Medical Sciences and Peking Union Medical College Beijing China; 2 Department of Geriatric Medicine Peking Union Medical College Hospital Chinese Academy of Medical Sciences and Peking Union Medical College Beijing China

**Keywords:** telerehabilitation, elderly, sarcopenia, resistance exercise, rehabilitation, gerontology, aging, randomized controlled trial, rehabilitation training, body composition, strength, balance, cardiorespiratory endurance, self-care, physical therapy

Thank you for your reading of our article “A 4-Week Mobile App–Based Telerehabilitation Program vs Conventional In-Person Rehabilitation in Older Adults With Sarcopenia: Randomized Controlled Trial” [1]. We are truly gratified that our study has garnered your attention and interest and has sparked meaningful discussion. In response to the points raised by the authors [2], our answers are as follows.

Regarding the first point, first, we had already fully considered that the older adult population might have difficulties in understanding movements accurately and that there could be differences in individual comprehension if they only watched videos and read the text. Therefore, before formally using the app for training, the telerehabilitation group attended an in-person session, where a physical therapist guided the participants through a simulation of the movements and provided detailed instructions on how to use the app. This was done to ensure that participants could follow our intended exercise protocols as closely as possible during home-based training and to minimize individual differences in understanding. These details are thoroughly described in the study methods. Second, the average age of the participants in the telerehabilitation group was 70.47 years. For feasibility, the study included patients who had basic literacy skills and were able to operate smartphones. We believe the study results are applicable to the population defined by the inclusion and exclusion criteria, but we do not claim that they can be generalized to all older adults with sarcopenia. How to support older adults with sarcopenia who cannot use app-based resources is an area worthy of further investigation. We agree that integrating motion-tracking technology into the study protocol to provide real-time remote corrections and guidance during telerehabilitation would be an ideal approach [3], which could be a direction for future improvement.

Regarding the second point, we agree that the effectiveness of telerehabilitation is related to the chosen setting. Results obtained in a specific hospital environment are suitable for generalization to similar contexts. How to assist older patients in areas with limited technological infrastructure is an important area for further research. Before the study, we had thoroughly considered the potential impact of comorbidities and medications on the study outcomes. We excluded patients with other neuromuscular diseases, those with severe skeletal disorders, or those using corticosteroids, which might confound the interpretation of the results.

Regarding the third point, muscle function is indeed influenced by a wide range of factors [4]. In addition to exercise, which is the most important factor discussed in this study, nutrition, sleep, and daily activity levels also play significant roles. It would be ideal to include all these factors in a single study. However, monitoring any of these indicators in the older adult population would add another layer of complexity to the research. We agree that mental health and social functioning should also be considered as end points [5], which may be further explored in future studies.

We truly appreciate the authors’ letter, which has prompted deeper reflection on our study and provided inspiration for future research directions.
